# The Emergence of Spindles and K-Complexes and the Role of the Dorsal Caudal Part of the Anterior Cingulate as the Generator of K-Complexes

**DOI:** 10.3389/fnins.2019.00814

**Published:** 2019-08-07

**Authors:** Andreas A. Ioannides, Lichan Liu, George K. Kostopoulos

**Affiliations:** ^1^Laboratory for Human Brain Dynamics, AAI Scientific Cultural Services Ltd., Nicosia, Cyprus; ^2^Neurophysiology Unit, Department of Physiology, School of Medicine, University of Patras, Patras, Greece

**Keywords:** sleep, K-complexes, spindles, magnetoencephalography, magnetic field tomography, error related negativity

## Abstract

The large multicomponent K-complex (KC) and the rhythmic spindle are the hallmarks of non-rapid eye movement (NREM)-2 sleep stage. We studied with magnetoencephalography (MEG) the progress of light sleep (NREM-1 and NREM-2) and emergence of KCs and spindles. Seven periods of interest (POI) were analyzed: wakefulness, the two quiet “core” periods of light sleep (periods free from any prominent phasic or oscillatory events) and four periods before and during spindles and KCs. For each POI, eight 2-s (1250 time slices) segments were used. We employed magnetic field tomography (MFT) to extract an independent tomographic estimate of brain activity from each MEG data sample. The spectral power was then computed for each voxel in the brain for each segment of each POI. The sets of eight maps from two POIs were contrasted using a voxel-by-voxel *t*-test. Only increased spectral power was identified in the four key contrasts between POIs before and during spindles and KCs versus the NREM2 core. Common increases were identified for all four subjects, especially within and close to the anterior cingulate cortex (ACC). These common increases were widespread for low frequencies, while for higher frequencies they were focal, confined to specific brain areas. For the pre-KC POI, only one prominent increase was identified, confined to the theta/alpha bands in a small area in the dorsal caudal part of ACC (dcACC). During KCs, the activity in this area grows in intensity and extent (in space and frequency), filling the space between the areas that expanded their low frequency activity (in the delta band) during NREM2 compared to NREM1. Our main finding is that prominent spectral power increases before NREM2 graphoelements are confined to the dcACC, and only for KCs, sharing common features with changes of activity in dcACC of the well-studied error related negativity (ERN). ERN is seen in awake state, in perceptual conflict and situations where there is a difference between expected and actual environmental or internal events. These results suggest that a KC is the sleep side of the awake state ERN, both serving their putative sentinel roles in the frame of the saliency network.

## Introduction

Decades of research have established a resilient sleep architecture, fairly reproducible electrophysiological features characterizing the sleep stages and impressive links between sleep macro- and microstructure with very important brain and body functions. Yet, key features of the brain mechanisms dictating these reproducible processes still escape our understanding (Kryger et al., 2017). Here, we focus on two of the most prominent of features of sleep microarchitecture, primarily on the K-complex (KC) and to a lesser degree on spindles. Eight decades after its first description (Loomis et al., 1938), the KC continues to attract the attention of researchers (de Gennaro et al., 2000; Colrain, 2005; Halász, 2005, 2016), because KC appears to be involved in significant sleep functions and several intriguing questions arose in the efforts to understand its underlying mechanisms (Loomis et al., 1938; Roth et al., 1956; Bastien and Campbell, 1992; Amzica and Steriade, 2002; Colrain, 2005; Halász, 2005; Wennberg, 2010; Jahnke et al., 2012).

The KC is a multicomponent event. The main and characterizing component is a large abrupt onset negativity in the electroencephalogram (EEG) (>0.5 s and up to 0.3 mV), often followed by a longer lasting positivity, while a shorter positivity preceding the negative wave may not always be discernible by eye (Cote et al., 2000; Colrain, 2005; Rodenbeck et al., 2006). Interpretation of some of these waves is easier in the case of sensory evoked KCs: Laurino et al. (2014) identify three peaks, or waves distinguished best by their latency (in msec): an early short positive P200, a large negative N550 and a late and longer lasting positive P900. The evoked P200 appears to be a specific sensory response acting as a traveling cortical excitation inducing the bistable cortical response (P550/900) response. A KC is often preceded and is usually followed by spindles (Kokkinos and Kostopoulos, 2011). Furthermore, the KC is a large physiological EEG event with widespread brain topography, its power maximizing frontally, which, along with spindles, characterizes NREM2 stage of sleep (Colrain, 2005). It often emerges at times of brain instability and it is considered both reactive, since it can non-specifically follow sensory responses to various stimuli, but also promote sleep, since it is fairly generalized.

The great practical value of KC is its use to unequivocally mark sleep onset, along with spindles, a demand for all quantitative studies of sleep in health and disease. Beyond that, several important roles for KCs in brain functions have been proposed by both animal experiments (Amzica and Steriade, 2002; Marini et al., 2004; Ji and Wilson, 2007; Cruz-Aguilar et al., 2015) and human brain metabolism (Larson-Prior et al., 2009; Maquet, 2010; Caporro et al., 2012; Jahnke et al., 2012) and EEG/MEG electrophysiological studies (Lu et al., 1992; Numminen et al., 1996; Yoshida et al., 1996; Murphy et al., 2009; Wennberg and Cheyne, 2013; Ioannides et al., 2017). One of these roles of KC is a sentinel role protecting sleep by preventing waking to normal stimuli, which do not present a danger (Jahnke et al., 2012; Halász, 2016). Another suggested function of KCs is to aid the homeostasis of synaptic activation and some stages of memory replay and consolidation (Tononi and Cirelli, 2006; Ji and Wilson, 2007; Johnson et al., 2010; Staresina et al., 2015; Kryger et al., 2017). Through all such “housekeeping” of brain synapses, KCs may pave the way for the uptake of new information after wake-up. Finally, substantial deviations of KC density have been documented in association to aging (Kubicki et al., 1989), epilepsy (Halasz, 1981; Steriade and Amzica, 1998; Geyer et al., 2006; El Helou et al., 2008; Seneviratne et al., 2016), Alzheimer’s disease (De Gennaro et al., 2001, 2017) and schizophrenia (Ramakrishnan et al., 2012).

We reported about a decade ago how the progression of sleep stages can be studied in terms of changes in the brain’s background activity (quiet “core” periods, free from any prominent phasic or oscillatory EEG or MEG events), especially in the gamma band (Ioannides et al., 2009). More recently we focused on gradual changes upon sleep onset and as sleep progresses through stage non-rapid eye movement (NREM)-1 and NREM-2 as well as before and during spindles and KCs. Figure 4 of our previous work (Ioannides et al., 2017) shows in one view the large changes in the activity of midline sagittal areas of the brain throughout light sleep. First during “core” periods of light sleep (relative to the activity before sleep), and the smaller changes before and during the large graphoelements of NREM2. With sleep onset fairly widespread increase in low frequency spectral power is identified in NREM1 core periods that extend wider in NREM2 core periods. When the NREM2 core period was contrasted directly with NREM1 core period, modest increases were identified in the alpha and sigma bands in three frontal areas; the area just in front of the genu of the anterior cingulate cortex (ACC), which is often referred to in the literature as rostral ACC (rACC), showed most clearly. The comparison between the periods before spindles with the NREM2 core periods show focal increases in the rACC in the delta band. We have interpreted this sequence of changes, especially in rACC, as a sign of extreme precautions before spindles proceed with their putative memory consolidation task (Ioannides et al., 2017). The apparent precautions before memory consolidation may be important, because memory consolidation during sleep may involve changes in the neural representation of self that is represented by the two midline areas found to consistently increase their gamma band spectral power from awake state to light, deep and finally REM sleep (Ioannides et al., 2009). This midline self-representation core (MSRC) consists of two distinct brain areas; the first, MSRC1, is on the dorsal medial prefrontal cortex and the second, MSRC2, is in the precuneus in the midline posterior parietal cortex (Ioannides, 2018).

The results reported in our earlier work (Ioannides et al., 2017), emphasized spindles because the main motivation was to provide a framework for understanding the role of sleep in learning (Ioannides, 2017) and the neural representation of self (Ioannides, 2018). The sequence of changes from awake state to NREM1 and NREM2 and in particular during the periods before spindles were consistent with preparatory steps designed to ensure accurate and uncorrupted memory consolidation during spindles that related to changes in the neural representation of self. In the earlier work (Ioannides et al., 2017), changes were identified before spindles that seemed to serve a sentinel role, a role better served by the KCs, the question why there is a need for two sentinel operations, with similar, yet distinct patterns of activations before spindles and KCs was nor addressed. Another key unresolved question of the 2017 paper was how the patterns of activation differentiate before spindles and KCs and in particular whether one or more areas could be seen to play a key role in the generation of KCs. To address these questions, we proceeded in this work using the same methods as the ones used in Ioannides et al. (2017), but analyzed at finer detail the more caudal and dorsal areas to the ones we emphasized in Ioannides et al. (2017). These areas are in the pre-motor cortex and its immediately ventral anterior cingulate cortex (ACC). These areas were not emphasized in the (Ioannides et al., 2017) study because they were swamped by the extensive changes over a wider area during KCs and the KC changes were not central to the discussion. In the current study the results are presented over the frequency ranges from 3.2 to 16 Hz and separately for the periods before and during spindles and KCs for the key areas in the medial frontal cortex identified from the new current analysis and the earlier studies (Ioannides et al., 2009, 2017; Ioannides, 2018). The new analysis anchors the description to the spectral changes before and during spindles and KCs in four nearby areas: the two subdivisions of the premotor cortex and the two areas directly ventral to them on the dorsal caudal ACC (dcACC) that we will refer to as dcACC1 and dcACC2.

Here we will focus on the changes before and to a lesser extend during KCs. Just like in our previous study we find that the controlling role is played by a set of areas in the ACC, and in the overlying ventral premotor areas. We will develop our description around the spectral changes before and during spindles and KCs in two nearby areas on the border of dorsal caudal ACC (dcACC) that we will refer to as dcACC1 and dcACC2 and the ventral premotor areas directly above them. The first area, dcACC1, seems to be involved in the generation of KCs. The second area, dcACC2, is the area of common increase in delta power before spindles and KCs. Our results provide a hint on how the uncertainties in our current understanding of KCs might be resolved and support our earlier results (Ioannides et al., 2017) and the results from other recent studies, claiming that KC-related activity is more prominently identified in frontal cortical areas (Colrain, 2005; Halász, 2005) along the cingulate gyrus. Critically, our results are in full agreement with the first demonstration of focal stimulation, while awake, of a specific cortical area, which seems to correspond to our dcACC1, evoking activity resembling KCs (Voysey et al., 2015). Our results, when seen in the context of numerous other studies of sleep and awake state, suggest that similar mechanisms and networks with key hubs in the ACC deal with pain, saliency detection and environmental monitoring during both sleep and awake states. This supports the suggestion made in our earlier study (Ioannides et al., 2017): the mechanisms that operate in sleep are similar and involve the same networks serving similar roles in awake state. As a consequence, our results are relevant not only for KCs and sleep, but also for fundamental questions about the role of ACC in awake state. We will comment in the Discussion session on how our results may contribute to the current debate about the way emotion, pain and cognitive control are processed in the ACC (Shackman et al., 2011; Jahn et al., 2016).

Our tomographic analysis allows the generators to be throughout the brain. In our description of the results we have focused more on structures close to a midline sagittal section, motivated partly by our own results, earlier studies (Ioannides et al., 2009, 2017) and by the results of the current study that also show distinctive changes on the medial walls of the two hemispheres. It was also motivated by the findings of many past studies reviewed and meta-analyzed in relation to movement and cognition that relate findings about activity patterns close to the medial walls of the two hemispheres both in primates and in human neuroimaging (Picard and Strick, 1996, 2001), related to conflict processing of both cognitive and emotional type (Bush et al., 2000), self-monitoring (Dehaene, 2018), negative affect, pain and cognitive control (Shackman et al., 2011; Jahn et al., 2016). All these studies show distinctive changes on the medial walls of the two hemispheres and this is where we focus the presentation of results. We also show enough of the results along coronal and axial cuts through the identified areas to convey to the reader the full richness of the results, the laterality and which activations are focal and which are extended.

## Materials and Methods

### Background of the MEG Studies

The experiment demanded a number of innovations to make possible, for the first and to the best of our knowledge still the only, set of whole night measurements in the MEG environment. Some details are provided in Ioannides et al. (2004) and its supplementary data and further details in Ioannides et al. (2017). Briefly, to achieve whole night MEG measurements at the time of the experiment (2000), new elements had to be introduced in the hardware of MEG, the data storage capability, the supine arrangement of the recordings and the ease of connecting and disconnecting the subject from the recording system if needed (e.g., to use the toilet in the middle of the night). The last modifications also demanded re-checking/calibrating after the subject returned for the continuation of the recordings. To ensure that all the changes worked well individually and with good synergy the final test was performed “from the inside” by one of us (AAI), who was not a good sleeper so these tests were usually performed after an overseas travel so that AAI was sleep deprived and therefore easier to sleep. On two occasions AAI stopped the recordings early and asked for modifications. On the third occasion (about a year after the start of the preparations for the sleep experiment) AAI slept for the entire night and gave the OK for the experiments to go ahead.

### Subjects

The experiment was conducted in RIKEN with RIKEN’s ethics committee approval. Before the experiment, all the subjects gave their informed written consent after all procedures were explained to them. Candidate subjects were carefully screened with selection criteria that included regular sleep habits, male, right handed (Annett, 1967) and free of neuropsychiatric illness and all medication. After the set up was ready for recordings and over a period of about a year, the six subjects who passed all selection criteria were invited to the lab and performed the planned procedures for the whole night sleep MEG experiments. The first night began with training and placement of auxiliary channels before the subject underwent the sleep acclimatization sleeping on a replica of the MEG bed and with his head inside a replica of the MEG helmet. The next morning the subject was waken up (after 8 h sleep if he had not already waken up) and after debriefing was released and worked as usual with the instruction to stay awake throughout the day. The subject returned to the laboratory after work, had an evening meal and was prepared for the recordings of the main MEG sleep experiment. The data from all seven subjects were scored offline by two sleep experts (six subjects selected and AAI who also satisfied the criteria except for the one for good sleep habits and the fact that he was sleep deprived at the time of the experiment). The two independent scores were compared, a common one agreed and the night hypnogram constructed. For each sleep stage, segments were identified with acceptable signal: free of noise segments, artifacts and good quality signal for MEG, EEG and all auxiliary channels and no indication for head localization coil movement, with head movement below 5 mm between successive head localizations measurements (obtained every 3 min). These conditions were satisfied for three of the six subjects and AAI. The data from these four subjects were prepared for the detailed tomographic analysis. Table 1 lists for each of the four subjects the age at the time of the experiment and the number of core POIs and good spindles and KCs identified (age 25, 30, 31, and 49).

**TABLE 1:** Subject age at the time of the experiment and number of events (KCm = multiple KC).

**Subject #**	**Age at the time of experiment**	**Core conditions (control)**			**EEG graphoelements for NREM2**		
			
		**ECW**	**NREM1**	**NREM2**	**Spindles**	**KC1**	**KCm**
(1)	25	8	8	21	12	8	23
(2)	30	9	11	16	10	10	15
(3)	31	8	10	11	9	11	14
(4)	49	8	9	26	8	9	11

### Data Acquisition and Processing

The MEG was recorded throughout the night using a 151 gradiometer whole-head system (CTF/VSM Omega System, Canada) at a sampling rate of 625 Hz and low pass filter at 208 Hz. The following auxiliary channels were recorded in synchrony with the MEG: scalp EEG from C3 and C4 locations referenced to A2 and A1, respectively, vertical and horizontal EOG and electromyogram (EMG) from the chin. The EEG and EMG channels were pre-processed independently of the MEG using filters appropriate for sleep scoring and/or event identification. Commonly used procedures were employed to score the sleep stages (Rechtschaffen and Kales, 1968). For details on sleep stage scoring and identification of core periods see Ioannides et al. (2004). The data were pre-processed and analyzed in the same way as reported in Ioannides et al. (2017), so only a brief summary of the main points will be given below, with more details provided at the end (see section “Biases in the Presentation and Interpretation of the Results”) outlining the logic for the selection of the results to best convey the key findings of the analysis in the context of voluminous related work in a wide range of a fields.

Spindles and single KCs were selected from within segments with clear NREM2 assignment. All periods of interest (POI) were selected by visual inspection of raw MEG data with POIs related to KCs and spindles selected well away from each other, from other graphoelements and noisy or undefined segments like transitions between sleep stages. The 2-s long “before” KC POIs was set for the 2 s segment ending about a 100 ms before the start of KC with no spindle or other large graphoelements within its duration or the 2 s before. The “during” KC POIs were defined as the 2 s beginning a few tens of milliseconds before the KC onset. Typically 8 to 20 KCs and spindles were selected for each subject from the whole night data. Finally, the best 8 (in terms of noise and absence of large amplitude EEGs (other than the KC) were selected for tomographic analysis. The results to be presented here use seven POIs in total: the quiet “core” periods of eyes closed waking (ECW), NREM-1 and NREM-2 and the periods before and during spindles, and the periods before and during KCs. We note that visual inspection alone would not distinguish POIs for NREM2 core periods from NREM2 POIs before spindle and before KCs. The only distinction between them is that the NREM2 core periods are well away, typically (a lot more than) 2 s, from spindles and KCs and any other large graphoelements (e.g., movement artifacts or micro-arousals). The choice of 2 s for the POI “during a KC” and “during spindles” was based on two considerations:

(a) the minimal duration to cover all EEG events. The duration of a KC starting from the initial positive peak and including the late positivity is 0.5−1 or more seconds (Colrain, 2005). Similarly for the POIs for the period “during spindles.” A spindle usually lasts for 0.5 to 1.5 s. Therefore 2 s would suffice for either of the events.
(b) the minimal time needed for an accurate power spectral analysis of signals as slow as 1 Hz; while remaining within relevant time limits.

For the rest POIs “before KC,” “before spindles,” and the core POIs a duration of 2 s was demanded for having the appropriate control for the comparison. The 2 s are also reasonable to capture a generator that precedes the large graphoelements, as recently demonstrated for alpha band activity (Feige et al., 2005): when the alpha component amplitude [extracted from independent component analysis (ICA)] was used as a regressor of the fMRI bold signal time course only signal decreases were found when the standard hemodynamic response function was assumed (with time delay of about 6 s). The alpha regressor revealed significant positive thalamic and mesencephalic correlations with a mean time delay of 2.5 s. This would be consistent with the true generator of alpha in the thalamus to precede the actual cortical alpha by a few seconds.

In our analysis we treat the core periods, i.e., a quiet periods with no graphoelements, as the foundation of each sleep stage. Conceptually, a core stay plays a role in our analysis, similar to that played by the ground state of a physical system in physics, i.e., a state of a system with the lowest possible energy. Typical examples are the ground state of atoms and nuclei. The core states of different sleep stages are distinct just like the ground states of different atoms are distinct. Specifically, the NREM2 core state is seen as the ground state from which POIs before spindles and before KCs can be “excited.” The fact that only increases in spectral power are encountered in the transitions from NREM2 core POI to either the POI before spindles or POIs before KCs adds more credence to the analogy of core states with ground states of atoms. The POIs during KCs are more extreme (energetic) events that are far removed from the core state and hence able to reach a wider range of excited states, something that fits well with the higher amplitude and more widespread increases identified during KCs.

### Source Reconstruction

Source analysis of MEG signals for each trial was performed using magnetic field tomography (MFT) (Ioannides et al., 1990; Taylor et al., 1999). For each subject the MFT analysis is performed using a source space fitted to the exact anatomical geometry (extracted from the MRI of the subject). The MFT input is the set of values for the available MEG channels at a single latency (relative to the onset of the stimulus or other time-marked event). These values may be taken from a single trial or from the ensemble average of a set of trials. The MFT output is a three-dimensional distribution of the current density vector field in the source space (i.e., the region of space where active sources can exist) and it is computed completely independently for each time sample of data. For each time sample of each single trial, the continuous estimate of the current density vector is sampled and stored at regular intervals, spaced about 8 mm across and refer to for simplicity hereafter as voxels. The MFT basic algorithm and the mathematical foundation of MFT is described in Ioannides et al. (1990, 2004), Taylor et al. (1999), Poghosyan and Ioannides (2008). Reviews of the MFT analysis and post-MFT statistical and connectivity analysis have been presented in Ioannides (2006, 2007). The majority of MFT studies until about 2010 were devoted in the analysis of MEG data obtained from well controlled stimuli and in particular visual stimuli placed in the quadrants of the visual field so the first entry to the cortical system could be clearly identified and compared to the expected part of the primary visual cortex (Tzelepi et al., 2001). This approach allowed nearly identical experiments to be performed on the same subjects in separate sessions with fMRI and MEG. In this way and using the fMRI localization as a gold standard, it was demonstrated that MFT could localize the activity with millisecond temporal resolution with accuracy of 3 to 5 mm (Moradi et al., 2003), which was as good as the accuracy of the location of the MEG sensors relative to the head, measured by the coregistration of the subject’s head in the MEG helmet and the anatomical MRI image of the subject’s brain.

The time domain analysis relies on the presence of a time-locking mechanism, usually provided by the onset of a stimulus, to align similar POIs with (sub-)millisecond accuracy. For the analysis of continuous data, e.g., resting state or sleep there is no such time-locking mechanism. For such cases we have first stayed with the time domain analysis and analyzed filtered data within a specific band (ensuring that 2−3 oscillations were present each time) (Ioannides et al., 2009) or carried out the statistical comparisons after the spectral power was computed for each voxel from the time-domain time series. In the second method of analysis frequency bands are used instead of time periods: a sliding window of frequencies (typically frequency band width of 3.2 Hz) is used to compare conditions or search for linked activity. We adopt here this second form of analysis, following exactly the same steps as described in detail in Figure 1 of Ioannides et al. (2017). The first stage of the analysis maintains the extraction of single trial, sample-by-sample tomographic estimates of activity, as it is done for evoked responses. Then we allow for the uncontrolled blurring in time by transforming each regional time course in the frequency domain using long (duration of 2 s) segments. The outcome of the transformation of the tomographic analysis in the frequency domain shows the spatial distribution of spectral power within a band of frequencies. The statistical comparisons are obtained from the MFT solutions by contrasting spectral power within a 3.2 Hz windows for the two POIs to be compared. The center of this window is initially placed at 3.2 Hz. The center of the window is then shifted by 1.6 Hz and an independent statistical comparison is performed for each new window. The width of 3.2 Hz is about the same as the width of frequency bands below 16 Hz and by stepping every 1.6 Hz we can look separately at the low and high parts of each of these frequency bands. In this way the sliding window center is from 3.2 to 94.4 Hz to cover the frequency range from 1.6 to 96 Hz (Table 2).

**FIGURE 1:**
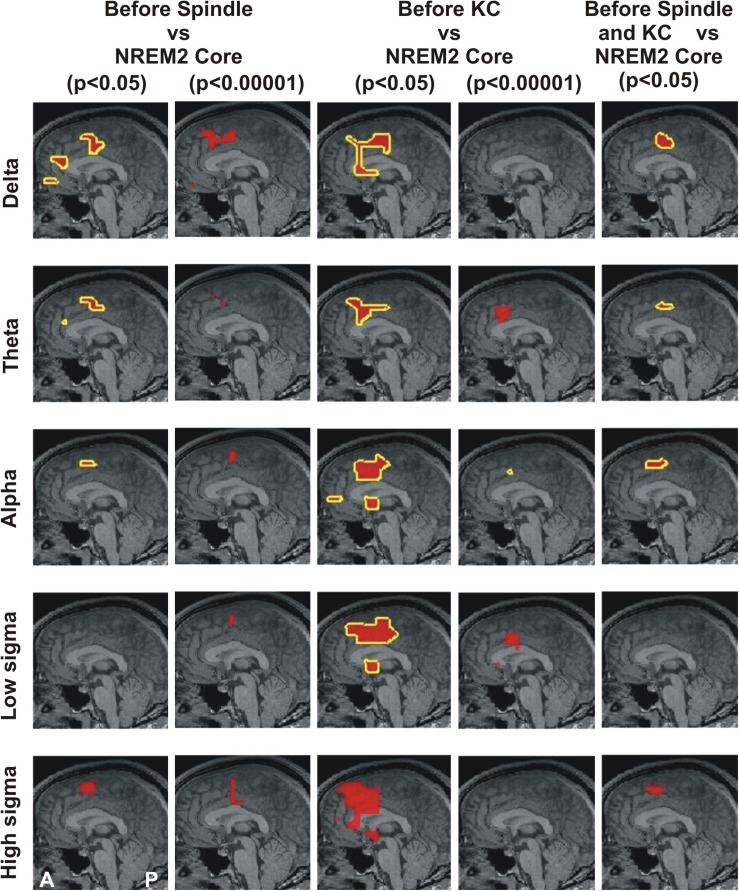
MEG derived tomographic estimates of increases in spectral power before spindles and KC compared to that during the NREM2 core periods. The increases are displayed if they are within 2 mm (on either side) of the displayed midline sagittal cut (Talairach coordinate x = 0). To link the information central to this paper with that presented in Figure 4 of Ioannides et al. (2017), the figure is arranged in the same general way, with each row representing the same frequency band and each column showing the results for a specific combination of POI comparison and statistical significance. The yellow outline denotes common increase in all four subjects. We also display as a the red blob without yellow outline increases in 3 out of 4 subjects in the given comparison and statistical significance, provided there is no other increase with 4/4 subjects anywhere in the brain. The following contrasts are shown: columns 1 and 2 show increases before spindles versus NREM2 control at *p* < 0.05 and *p* < 0.00001; Columns 3 and 4 show increases before KCs versus NREM2 control at *p* < 0.05 and *p* < 0.00001, respectively. Column 5 shows the area with modest increase (*p* < 0.05) identified in separate statistical comparisons from non-overlapping POIs for the periods before spindles and before KCs. The same five frequency bands, as defined in Table 2, were used as in Figure 4 of Ioannides et al. (2017): delta (3.2 Hz), theta (6.4 Hz), alpha (9.6 Hz), low sigma (11.2 Hz), and high sigma (14.4 Hz).

**TABLE 2:** Definition of various frequency bands and sub-bands used in this paper.

**Frequency band name**	**Center in Hz**	**Range in Hz**
Delta	3.2	1.6–4.8
Transition delta/theta	4.8	3.2–6.4
Theta	6.4	4.8–8
Transition theta/alpha	8	6.4–9.6
Alpha	9.6	8–11.2
Low sigma	11.2	9.8–12.8
High sigma	14.4	12.8–16
Sub-band of High gamma	57.6	56–59.2

The EEG frequency band division has been arbitrarily chosen (Buzsaki, 2006), limited by available recording technology, often lacking consensus especially when comparing records at different developmental stage, vigilance state and species recorded. Therefore, in order to have a more complete view of developments in the frequency domain, we adopt a moderate resolution in frequency that can be referred easily to the (not so) standard traditional bands but it has a somewhat finer resolution and an absolute quantitative basis, as described in the previous paragraph. This slightly more detailed and extended range describes well traditional and transitional frequency bands (Table 2) and allows us to relate the new results with those of our earlier analysis (Ioannides et al., 2017). The emphasis is on the low frequencies below the beta range, with only one example of changes in higher (gamma) band. This was necessary to keep the size of the manuscript to reasonable length. The activations below the beta band deserve to be grouped together, while separate studies are needed to do justice to the beta and gamma bands. The single result in the gamma band is included to demonstrate the presence of significant changes in activity in the high gamma band and show the distinct pattern of changes (in terms of statistical significance) in the low (below beta) and high (above beta) frequencies.

#### Common Spectral Statistical Parametric Mapping (sSPM)

We will present results for changes that were identified with the same sign (increases or decreases) and satisfying the *p*-value statistical threshold for each one of the four subjects. All our *p*-values are corrected by the conservative Bonferroni correction for multiple voxel comparisons. No additional correction was made later because the conservative nature of the Bonferroni correction for the 1,000 or so voxels would have more than compensated for the few frequency bands used in the analysis. The spectral statistical maps of each subject were transformed to MNI template so that they could be combined across subjects and thus identify common changes in brain activity at predefined statistical thresholds. A new *t*-value was assigned in this common space by smoothing the original *t*-values within a sphere of radius 12 mm using a Wood Saxon kernel of radius 7 and decay constant 4 mm. This operation had two effects. First it allowed for possible errors in localization (which through segment selection from periods of very small head movement should be small, within a few millimeters), individual differences in anatomy, and inherent inaccuracy of transformation into a common space. Second it smoothed and further reduced the value of the *t*-test around the peak voxels, thus ensuring that high *t*-values in the common space had also a reasonable extend. The same two threshold levels of significance were used, as the ones used in the 2017 study (Ioannides et al., 2017): the lower significance threshold is set to *p* < 0.05 and corresponding changes will be referred to as “modest.” The second threshold is set at a much higher level of significance of *p* < 0.00001 and is correspondingly referred to as “prominent.” The grand sSPM results were finally computed, separately for each comparison between two POIs or conditions by counting for each voxel the number of subjects that showed increase or decrease of activity at a pre-defined threshold of *p*-value (always after Bonferroni correction for multiple voxel comparisons).

Automatic identification of contours of similar significance in the sSPMs are naturally blurred by the accuracy of the reconstructions, possible head movement, and particularly the operations for combination across subjects. The smoothing of solutions combines also with the tendency of the plotting routines to smooth over the source space points at which the sSPMs are computed. Nevertheless the shape of the contour is indicative of what the areas might be. When a single area passes the threshold then the contour is a tight spherical shape around the correct brain area. If two areas next to each other pass the threshold then a cylindrical shape will be extracted with the cylinder’s symmetry axis along the line joining the two nearby areas. For example if the supplementary motor area (SMA) and pre-SMA are both identified in the sSPMs then the automatic contour computation will produce a cylindrical solution containing both SMA and pre-SMA, while if SMA and dcACC2 are simultaneously satisfying the threshold set for the sSPMs the contour will have again a cylindrical shape but with the symmetry axis in the ventral-dorsal direction. If all three areas pass the statistical threshold and their (nearby) centers form a right angle then a right angle wedge will result (as we will see in some of our examples in the Results section. By exploring the sSPMs at different frequencies and at different statistical thresholds it then becomes possible to disentangle focal contributions from ROIs because the different areas have a different spectral behavior. Following this analysis we have identified seven key areas, four identified in this study, one in the 2018 study and two others identified in the 2017 study and again showing prominently in the new more detailed analysis.

The resulting grand sSPM results in the MNI space were then back-transformed to the anatomical space of one subject so they could be displayed in the background of that subject’s MRI. As a general rule, we will show results when the set statistical threshold (*p* < 0.05 or *p* < 0.00001) is satisfied by each one of the four subjects. In only one case (in Figure 1) we have adapted the presentation to show additional cases where the set statistical threshold is satisfied for 3 of the four subjects, provided there was no higher, i.e., 4/4 case anywhere else in the brain. Comparison at different statistical threshold has a specific interpretation in the case of tomographic solutions obtained with our analysis of MEG data. In the time domain increasing the threshold allows us to see events over finer time scales, e.g., to identify the first entry in V1 (Poghosyan and Ioannides, 2008). In the frequency domain identifying a change within a specific frequency band limits the options for neurophysiological interpretation (although details of the mechanisms can only be identified by different experiments).

In summary, we followed the same procedures for analysis and reporting the results so that the new results can be compared and seen in the context of our previous work (Ioannides et al., 2017), with only one exception. In the earlier work, we used the Talairach space as the common template to combine the data across subjects, while in this work we used the MNI space. The data in both works were back-transformed to the MRI of the same subject. This small difference allows a direct comparison whenever results from the same POI contrast and at the same statistical threshold are used. This comparison provides a measure of the distortions that transformation to a common space might introduce, in combination with the other operations (smoothing, interpolations etc.) for the second level (across subjects) statistics.

### Biases in the Presentation and Interpretation of the Results

We state from the outset that it is not possible to identify the mechanisms underlying activity in the delta band or fully define any particular EEG event on the basis of spectral power of background (core) activity alone (Amzica and Steriade, 1998). It is also true that the mechanisms underlying delta waves are still a matter of debate, i.e., regarding their emergence from thalamocortical or cortico-cortical circuits (Steriade and Amzica, 1998) and their characterization as type I or type II (Bernardi et al., 2018). The present MEG tomographic study can offer little help in understanding electrophysiological neuronal mechanisms beyond directing attention to particular brain areas with pivotal role in the circuits producing the examined EEG events. However, in an effort to provide a consistent framework to interpret the observed spectral power changes in terms of putative brain functions we considered local power in the delta band (in the sense of slow, 1−4 Hz) as reflecting hyperpolarizing potentials of neurons in the particular area (due to synaptic action, disfacilitation or any other effect (Cash et al., 2009)) and therefore consider the observed increase in delta power as a change toward inhibition. We nevertheless stress that we cannot on the basis of present data alone, commit to any one mechanism conferring bistability, beyond taking into account in our discussion that KC is viewed *as a momentary excursion of the cortex to an unstable state* (Wilson et al., 2006) *or as an evoked neural bistability* (Allegrini et al., 2015).

Upon sleep onset, alpha is blocked. Our finding is the reappearance focal prefrontal brain areas during NREM2 core periods (Ioannides et al., 2017). We consider this as an excitation; the term used at the level of brain functionality rather than at the neuronal neurophysiology level. The correlation of increases in EEG alpha to cognitive processes has long been demonstrated (Wolfgang, 1999), a long with various roles in different aspects of cognition attributed to theta and higher frequency bands activations (Başar et al., 2001). Alpha increases during creative cognition and this may reflect internal processing demands (Fink and Benedek, 2014). Specifically, increased alpha band activity in sleep was shown to correlate to increased sensitivity to environmental noise (McKinney et al., 2011). We therefore consider alpha band activations as indicative of partial return of cognitive processing lost at sleep onset. This leaves theta as a transition zone, possibly acting as a control frequency balancing the putative inhibition from delta and putative excitation from alpha.

The results of this work relate to studies in diverse areas of neuroscience where important definitions regarding locations in the brain and frequency bands are labeled in a fuzzy way, and often inconsistently by experts in different fields, or even within the same field. We compromise the conflicting requirements of relating our work with numerous studies in diverse fields and keeping specificity and accuracy in our presentation of the results by referring as much as possible to precise values of quantities rather than using human labeling and attributions for frequency ranges and brain areas. We computed spectral power and compared the distributions of spectral power for different POIs within frequency bands of fixed width of 3.2 Hz, and we quoted the results for such bands referring to the center frequency (in Hz) of the band. We refer to spatial locations in terms of Talairach coordinates (TC) (Talairach and Tournoux, 1988). When the coordinates that we refer to from other studies are given in the MNI space, we translate them to TC using the Yale BioImage Suite Package^1^. Wherever appropriate, we refer to the popular (but not always consistent) naming of frequency bands and brain locations, also providing enough information for the reader to refer to the exact spatial location in TC.

### Region of Interest Identification and Interpretation of the sSPMs

In this work we make an indirect region of interest (ROI) analysis using the seven areas defined below.

The first ROI is the frontal area increasing in gamma band from ECW through light and deep sleep, reaching its highest activity during REM (Ioannides et al., 2009); this area was identified as the frontal component of the medial self-representation core (MSRC1) in Ioannides (2018). The second area is the rostral anterior cingulate cortex (rACC). Changes were identified throughout light sleep in rACC consistent with a sentinel role, particularly in the POI before spindles. These changes were interpreted in our previous study as stopping external and internal influences before spindles (Ioannides et al., 2017).

In this work we pay more emphasis on the details of activations in the pre-motor cortex and the immediately ventral part of ACC. We are able to disentangle changes in at least four distinct brain areas by looking in detail in the frequency range from delta to high sigma, before and during spindles and KCs, and for two levels of statistical significance. After transformation to a common space it is clear that these four areas correspond to the distinct cytoarchitectonic areas SMA proper and pre-SMA of the premotor cortex and two other areas directly ventral within the dorsal caudal part of the ACC, which we name dcACC1 and dcACC2. These areas are likely to correspond to two distinct cytoarchitectonic areas in this part of ACC, each with its own somatotopy (Picard and Strick, 1996, 2001).

Finally, the seventh area is in the in the sub genual ACC (sgACC) at the ventral anterior edge of the widespread prominent changes during KCs and hence not easily differentiated from it in the earlier work (Ioannides et al., 2017). It is also identified as a more focal modest increase in the pre-KC POI.

## Results

### Event Count and Time Domain Analysis

In agreement with the reports for spindles (Frauscher et al., 2015) and KCs (Mak-McCully et al., 2015), for each and every subject we studied, our own tomographic estimates of activity extracted from MEG signals of individual spindles and KCs also show widespread and asynchronous peaks in the time domain and widespread increases in spectral power over the delta, theta and alpha bands. These findings justify our analysis being deliberately insensitive to precise timing. We therefore proceed with detailed analysis of the sSPMs.

### Changes Before and During Spindles and KCs Relative to NREM2 Core Periods

Figure 1 displays the common changes across subjects in spectral power (relative to NREM2 core) in the mid-line sagittal cut. For ease of comparison it is set in a similar format as Figure 4 of our previous work (Ioannides et al., 2017) Figure 1 shows in separate columns the results for POIs before and during spindles (columns 1 and 2) and KCs (column 3 and 4), separating also in different columns results at *p* < 0.05 (columns 1 and 3) and *p* < 0.00001 (column 2 and 4). The fifth column displays the common changes across all subjects that survive the modest threshold of *p* < 0.05 for each subject and for before spindles and before KCs POIs. The Figure shows that only increases (relative to NREM2 core state) were identified. During the 2 s before spindles and KCs, only a single focal prominent activity increase survived with the statistical threshold set to *p* < 0.00001 in each and every one of the four subjects. This prominent increase over NREM2 core periods was only seen before KCs (not seen before spindles) and it was confined to a very small volume in the dcACC. This prominent pre-KC increase was present in all four subjects for only two of the bands, (of 3.2 Hz width): the displayed alpha band (center frequency 9.6 Hz) and the lower band with center frequency at 8 Hz (not displayed). It is the activity within and around this area that grows during the KC, in both intensity and extent and it covers more brain area and a wider range of frequencies. Each figurine displays in red surrounded with a solid yellow contour the regions where all (4/4) subjects satisfy the set criterion. When 3 out of 4 subjects satisfy the set criterion and there is no higher prevalence (i.e., 4/4 subjects) a red blob is displayed with no contour. This representation allows a comprehensive description of the increases in spectra power conveying information about prevalence amongst subjects, frequency bands and spatial location, while maintaining the sharpness of the main result: changes in spectral power before the large NREM2 graphoelements (compared to NREM2 core) are identified with high statistical significance in only one very focal prominent increase in a narrow range of frequencies and only before KCs.

**FIGURE 2:**
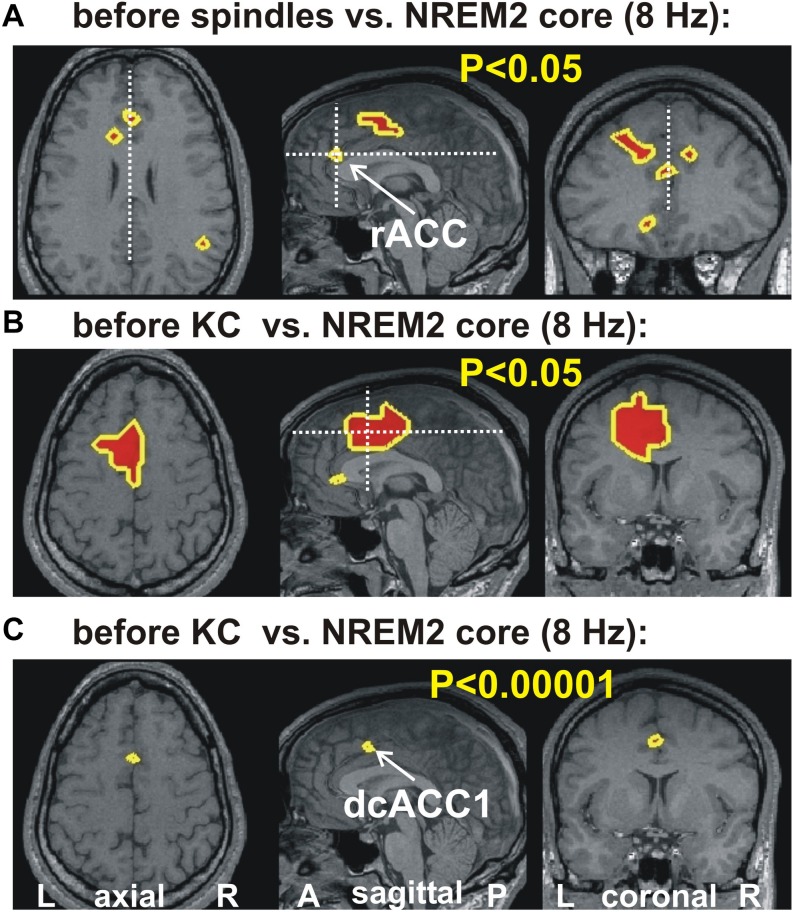
The key areas showing significant change in spectral power, common for all four subjects, at 8 Hz, from the comparison between before spindle, before KC with the NREM2 core POIs. The first two parts show the foci with modest (*p* < 0.05) increase along the midline sagittal cut and through the key area for before spindle **(A)** and before KC **(B)** POIs. **(A)** the axial and coronal cuts are through the rostral ACC area (rACC), which appears to play a key role in controlling the emergence of spindles (Ioannides, 2017). No common increase was identified for the before spindle period at the “prominent” threshold of *p* < 0.00001 in any one frequency band. **(B,C)** show, respectively, the modest (*p* < 0.05) and prominent (*p* < 0.00001) increase for the before KC POIs. In **(B)** a wider but still fairly compact area is seen at more caudal and dorsal along the cingulate; a second very focal increase is identified in the dorsal part of the subgenual ACC (sgACC). In **(C)** only one tiny area survives, and persist into the next frequency band with center at 9.6 Hz (see third row third column in Figure 1). The axial and coronal views in both **(B,C)** are through this area which we refer to as dcACC1.

Figure 2 shows the increases in activity relative to NREM2 core periods at 8 Hz in three orthogonal views. Modest increases are shown in parts A (before spindles) and B (before KCs), with part C showing the only prominent focal increase before KCs when the threshold is set at *p* < 0.00001 (no prominent increases are identified before spindles).

**FIGURE 3:**
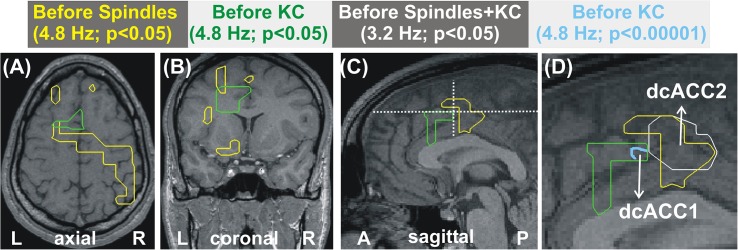
Distinct patterns of increases in spectral power for before spindle and before KC POIs over the NREM2 core periods. The increases are represented as outlines with the color of each outline corresponding to a different contrast as described in the text printed with the same color in each of the panels at the top of the Figure, and described also in this caption below. **(A–C)** show the areas with modest increase (*p* < 0.05) at the center frequency of 4.8 Hz for spindles (yellow) and KCs (green). **(A,B)** are the respective axial and coronal view cut along the lines in the mid-sagittal view in **(C)**. **(D)** shows a zoomed copy of **(C)**, with the focal area in cyan outline showing prominent (*p* < 0.00001) increases before KCs. The white outline marks the areas showing the common overlap for modest increases before spindles and before KCs in the slightly lower band (center at 3.2 Hz). The white outline marks much of the area with before spindle increase because the before KC increase at 3.2 Hz covers almost all of the area of the pre-spindle increase and extends well beyond. The centers of the cyan and white outlines are referred to as dcACC1 and dcACC2.

The increases in spectral power before spindles and KCs have distinct spatial and spectral properties. In the delta band (center frequency 3.2 Hz, range 1.6 to 4.8 Hz), the increases in spindles and KCs have some overlap but the areas can also be identified where even modest increases are evident in one but not the other condition. This may simply due to the fact that in one contrast increases have not reached the threshold for significance for all subjects. A more careful examination of the data suggest that the changes over NREM2 are very specific in their spatial and spectral details, with spectral power in a specific brain area changing in a characteristic way for spindles and in a different way in a nearby area for KCs. Figure 3 combines information from the earlier figures to demonstrate the evolution in the frequency domain from a common increase in the delta band in one brain area into distinct increases in nearby areas at the next higher frequency band. Figure 3C, shows that for the transition band centered at 4.8 Hz (from 3.2 to 6.4 Hz), there is a clear tendency of the modest increases in spectral power before spindles (yellow contour) and before KCs (green contour) to be localized in nearby but distinct, not overlapping areas. In Figure 3D, the mauve outline shows the very focal prominent increase at 8 and 9.6 Hz for the before KC POI. Finally the overlap region in the delta band where the POIs before both spindles and KCs show a modest increase relative to NREM2 core is shown by the white outline.

**FIGURE 4:**
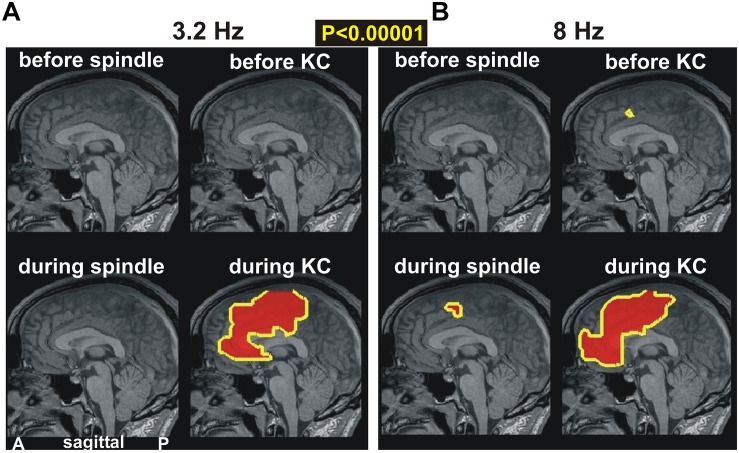
The key prominent changes in spectral power in midline areas for spindles and KCs at frequencies of **(A)** 3.2 Hz and **(B)** 8 Hz, respectively. Top rows show changes before periods while bottom rows for during periods. Left column of each figurine is for spindles while right column for KCs. A prominent broad increase is seen only during KCs in both frequency bands (bottom rows). The top row shows again the only prominent increase in the dcACC1 before KCs. A nearby prominent increase is seen during spindles and a much wider area during KCs.

In Figure 4, we examine prominent (*p* < 0.00001) changes in spectral power in the ACC and surrounding areas. Figure 4A shows changes in the lowest computed band (3.2 Hz) and Figure 4B at the higher band (8 Hz). The periods before spindles and KCs are displayed in the top row and reiterate the earlier result: the only prominent change in the pre-KC period is the increase in the high delta/low alpha range in the dcACC1. The periods “during” are displayed in the lower rows. During spindles there is a modest increase (not shown), but no prominent increase, in the delta band. A prominent increase is seen during spindles in the theta band that persists in the alpha and low sigma frequency ranges (not shown). What is striking is that this increase in prominent theta, alpha and low sigma bands during spindles maintains the spatial extent (as shown in the left, lower figurine for Figure 4B) and surrounds on the caudal and dorsal sides the area of prominent increases before KCs (dcACC1), but has no overlap with it, even when the modest threshold is used. By far the most dominant increase of activity is identified during KCs along the anterior part of the ACC for the entire range of delta, theta (seen in Figure 4B) and alpha bands (not shown).

### Changes in the Gamma Band Before and During KCs

**FIGURE 5:**
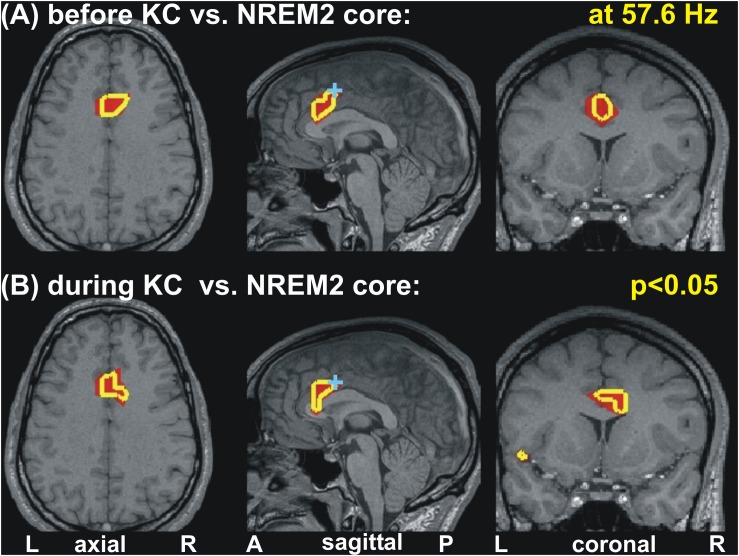
Changes of spectral power at 57.6 Hz before and during KC periods relative to the NREM2 core periods. Modest (*p* < 0.05) increases are seen in similar part of the anterior cingulate for the periods before **(A)** and during **(B)** KCs. A cyan cross in the mid-sagittal cut marks the area (dcACC1) showing prominent increase in upper theta and low alpha band seen before KCs. The axial and coronal cuts are through dcACC1.

Increases in spectral power are seen in the gamma band before and during KC POIs relative to the NREM2 core periods. Similar gamma band changes are identified for POIs before and during KCs, which, unlike the case in low frequencies remain focal and modest in both POIs. More specifically for gamma band changes, there was no prominent change (*p* < 0.00001) only modest ones (*p* < 0.05), identified within 3−5 successive bands (of width 3.2 Hz). These modest increases in activity before and during the KCs were in different parts of the ACC at different gamma bands. In some of the identified gamma bands, modest increases were identified at very similar locations for before and during KCs POIs. An example of such an increase in gamma band is shown in Figure 5, for center frequency at 57.6 Hz with a band width of 3.2 Hz: the area with modest increase is just rostral and ventral to dcACC1.

### Gathering Together the Results

**FIGURE 6:**
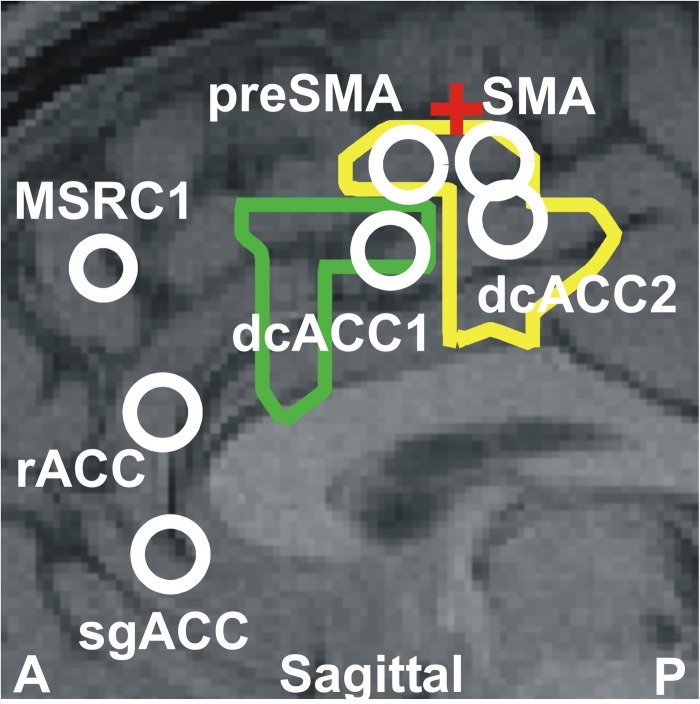
The seven key areas identified in this paper and in our previous studies (Ioannides et al., 2009; Ioannides, 2017); they are the six areas that play a key role in REM2 sleep and the area monotonically increasing its gamma power as sleep progresses from light to deep and finally REM sleep (Ioannides et al., 2009). The areas are displayed onto a mid-sagittal MRI about 2 mm inside the left hemisphere. The area dcACC1 plays a pivotal role in the generation of KCs. Three other areas are identified adjacent to dcACC1. One of these areas lies within dcACC; this area dcACC2 is just caudal to dcACC1 and is related more to spindles but it also plays a role for KCs, especially in the delta band. The area dorsal to dcACC1 is in the pre-SMA and the one rostral to dcACC2 in the SMA. These two areas are activated prominently (with dcACC2) during spindles and seem to make up the generator of the slow spindles. These two premotor areas show modest increases during both KCs and spindles in the theta and alpha bands. The two areas close to rACC seem to play a distinct role. The more dorsal area rACC relates more to spindles. The more ventral area relates to KCs and it is on the subgenual ACC (sgACC), at its more dorsal and ventral part close to the border with rACC. The two outlines showing the modest increase in activity at 4.8 Hz for spindles (yellow) and KCs (green) are also projected on the same MRI slice. The assignment of the two premotor areas to SMA and pre-SMA is made by reference to two earlier works early works for separating Broadman area 6 into SMA and pre-SMA sub-divisions. The first, relates animal studies to early human imaging studies of the areas (Picard and Strick, 2001) and the second is a recent work combining cytoarchitectonic and functional information (Ruan et al., 2018). The red cross marks the location of the rough border of SMA and pre-SMA as defined by the blue cross-hair in Figure 7 of Ruan et al. (2018). The TC of these areas and details of the spectral power changes for each one before and during KCs are given in Table 3.

The results described above together with the results of our previous two studies (Ioannides et al., 2009, 2017) highlight the critical role played by the ACC and the overlying cortex throughout sleep and specifically in the generation of KCs. The way this role is played out depends critically on changes in spectral power in very specific cortical locations at specific frequency ranges. Figure 6 displays the cortical geography on the midline cut with the seven key frontal lobe areas, located in either on, or within a few millimeters of the midline sagittal plane. In Table 3, we provide for each of the 7 key areas the full details about the increases relative to the NREM2 core periods for all bands up to 17.4 Hz, with center frequency starting at 3.2 Hz stepped by 1.6 Hz till 16 Hz. We emphasize that although there is a sharp transition around 16 Hz, with considerably lower spectral power at higher frequencies, changes are nevertheless seen at higher frequencies, especially in the gamma band as demonstrated by the results in Ioannides et al. (2009) and the example in Figure 5.

**TABLE 3:** A complete representation of the variation in spectral power of the seven midline areas (displayed in Figure 6) in the frontal lobes that play a key role before and during spindles and KCs.

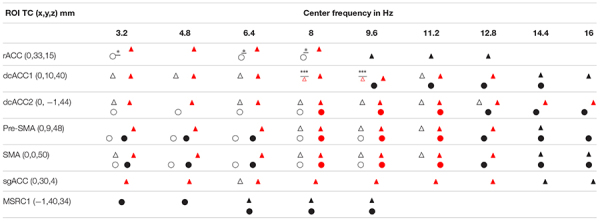

Each column shows the results for a spectral windows starting from 3.2 and ending at 16 Hz (window width of 3.2 Hz and step of 1.6 Hz). Each row represents a different ROI. The first area rACC is discussed in Ioannides et al. (2017). The last area is the center of the edge-surface of MSRC1 (Ioannides et al., 2009; Ioannides, 2018) closest to the midline. The remaining five areas related to the work described in this paper, but some also featuring in the results of Ioannides et al. (2017). Different symbols represent each graphoelement (triangles for KCs and circles for spindles) and POI (open symbols for POIs before and filled symbols for POIs during graphoelements). For ease of reference to POI, the open and filled symbols are placed on the left and right of each cell, respectively. Color is used to represent statistical significance, with black representing modest increases (*p* < 0.05) and red representing prominent increases (*p* < 0.00001). In addition we also mark for emphasis the only prominent increases in the before POIs (only for KCs) with ^∗∗∗^ and with ^*^ the rACC increase in low frequencies before spindles. The contrasts are always between a given POI related to a graphoelement with the NREM2 core period. The rACC is actively inhibited before spindles (shows a modest increase in the delta band) and in the frequency bands centered at 6.4 and 8 Hz. The key area identified in this paper is dcACC1. This area shows the most persistent increase in spectral power before KCs: the only area (i.e., only open red open symbols) with prominent increase in spectral power in a POI before NREM2 graphoelements. This increase is confined to the spectral windows with centers at 8 and 9.6 Hz (the two open red triangles in the dcACC1 row). Before KCs, modest increases are also identified in dcACC1 at lower frequencies (centers from 3.2 to 6.4 Hz) and at one higher band (center at 11.2 Hz). During KCs, dcACC1 shows prominent increases in all bands from 3.2 to 12.8 Hz and modest increases at 14.4 and 16 Hz. Before spindles, dcACC1 shows no increase in spectral power at any frequency band. During spindles dcACC1 show only modest increase for bands with centers from 9.6 to 14.4 Hz. The increase in dcACC2 is almost identical to dcACC1 before and during KCs, without reaching prominent level before KCs. The activity of dcACC2 differs markedly around spindles. Before spindles, dcACC2 shows modest increase in all frequency bands from 3.2 to 8 Hz, and no significant increase at higher frequency bands. During spindles, there is no significant change in activity in the lower bands (with centers at 3.2, 4.8 and 6.4 Hz) and a prominent increase in the next three bands (centers at 8, 9.6, and 11.2 Hz) and modest increases at the higher bands (centers at 12.8, 14.4, and 16 Hz). The changes in spectral power are identical for pre-SMA and SMA for both before and during POIs for spindles and KCs for all bands with centers between 4.8 and 14.4 Hz. The similarity persists in the lowest and highest bands, with only two differences: before KCs at 3.2 Hz (black open triangle) and during KCs at 16 Hz (black filled triangle) resepectively, pre-SMA does not show the modest increase that is evident in SMA. The sgACC shows modest increase in spectral power before KCs, only at the band centered at 8 Hz. Both rACC and sgACC show a focal increases at 8 Hz: rACC before spindles and sgACC before KCs. During KCs both areas show prominent increases in the frequency bands centered from 3.2 to 8 Hz. In the next three higher bands (centered at 9.6Hz) the sgACC continues to show prominent increase but for rACC only modest increases are identified. For sgACC the last two bands, centered at 14.4 and 16 Hz show modest increase. The seventh, most rostral and dorsal area, does not show increase in spectral power before either spindles or KCs. During spindles, it shows modest increase in spectral power at all bands with centers from 3.2 to 11.2 Hz and during KCs modest increase at bands with centers 8, 9.6 and 11.2 Hz.

**FIGURE 7:**
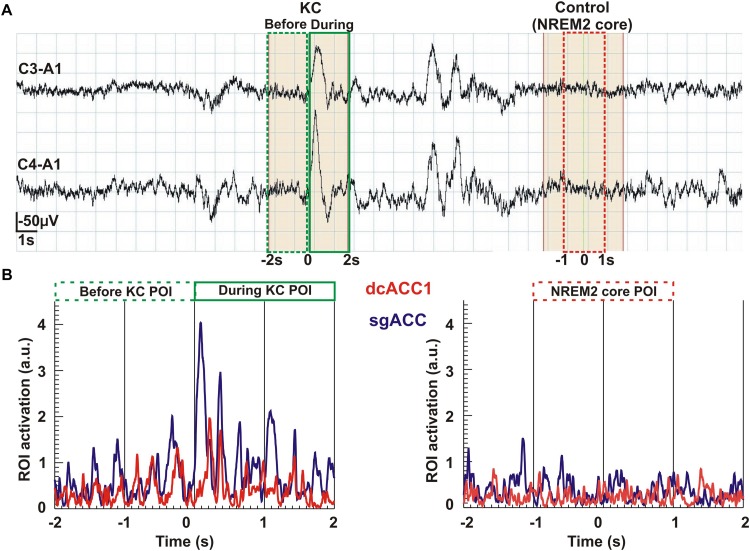
An overview of the analysis from the raw signal and POI selection to regional time courses. The top row Part **(A)** shows the raw EEG signal (high pass at 0.2 Hz) recorded from the two pairs of electrodes (C3-A1 and C4-A1). In the continuous, 37 s long NREM2 record, two KCs are identified. From this segment of data we selected for further analysis the first (single) KC and a period of NREM2 core, at least 2 s after the second (multiple) KC and with no other spindle or KC after its end for the remaining 6 s of the displayed segment. The beginning of the KC as it appeared in the lower of the two EEG channels was used to mark the middle of a 4 s long segment extracted for further analysis. We applied MFT tomography to analyze independently each one of the 2,500 samples of the single KC and each one of the 2,500 samples of the NREM2 core. We used for the Fourier analysis the 1250 samples in each of three POIs: the first 2 s of the KC defined the pre-KC POI and the last 2 s the during-KC POI. The middle 2 s of the NREM2 shaded 4 s were used as a NREM2 core POI for the further analysis. The spectra for each source space point (about 1000 active points in the source space spread in a uniform grid within the brain) was then computed from the time course of each POI. The sSPM were computed independently for each source space point from the statistical comparisons between the distributions for two POIs, each with eight exemplars for each POI. The results were reported after the conservative Bonferoni correction for multiple source space points was applied. Part **(B)** shows the time courses for modulus of activity (time-domain description) for the two ROIs (dcACC1 and sgACC) identified in the sSPM statistics as showing prominent changes before and/or during KCs relative to NREM2 core.

In the final display (Figure 7) we show time courses of KC and NREM2 core. The top part, Figure 7A, shows the time courses of the EEG signal, showing how the 2 s POIs used in the sSPM analysis are defined in the 4 s long segments extracted for the tomographic analysis. The lower part, Figure 7B shows the time courses for regional brain activations before and during the KC on the left and during the (4-s long) NREM2 core period. Part B shows the traces for the two ROIs linked more strongly to KCs, the ROIs for dcACC1 and sgACC. The traces provide a glimpse of the richness in the signal and the tomographic solutions derived from it.

## Discussion

### Summary of the Results and Their Relevance to Previous Studies

The main contribution of this paper is the delineation of increases in activity in four nearby brain areas before and during spindles and KCs relative to the NREM2 core periods. Two of these areas are in the cingulate cortex, dcACC1 and dcACC2 and the other two are located directly dorsal in the pre-motor cortex, corresponding to its two sub-divisions, the pre-SMA and SMA, respectively. The changes in each of these four areas is specific, complex and principled for the pre- and –during POIs of spindles and KCs. A modest (*p* < 0.05) increase in dcACC1, relative to NREM2, is seen for 3.2 Hz wide bands centered in all frequencies from 3.2 to 11.2 Hz, but this increase reaches prominent level (*p* < 0.00001) only for two bands, centered at 8 and 9.6 Hz. These two prominent increases in dcACC1 in the pre-KC POI, are the only prominent increases identified throughout the brain in the POIs before either spindles or KCs. For spindles no significant change in dcACC1 activity is seen in the pre-spindle POI and modest increases during spindles. The more caudal area dcACC2 shows modest increases relative to NREM2 core POI, for both the pre-spindle and pre-KC POIs for frequency band centers 3.2, 6.4, and 8 Hz, but only for spindles at 6.4 Hz and only for KCs at 9.6, 11.2, and 12.8 Hz. During the POIs before spindles and KCs the changes in SMA and pre-SMA are similar to the pattern of increases dcACC2. Table 3 provides the more comprehensive description as it puts together the new findings (emphasizing the POIs before spindles and KCs and focusing more on the KCs) and the earlier results describing mainly focal changes for spindles and emphasizing the POIs during spindles and KCs.

In comparing the here presented Figure 1 with Figure 4 in Ioannides et al. (2017), the two methodological differences of section 2.5 should be noted (use of MNI rather than TC for intermediate common space and allowing also the display of results satisfied by 75% of subjects). This direct comparison is possible for the first and third columns in Figure 1 which correspond to the fourth and sixth columns, respectively, of Figure 4 of the 2017 work (Ioannides et al., 2017), because for these cases exactly the same contrast and threshold are used in the displays. The second difference allows a more comprehensive description to be given for the highly focal activations before KCs that make up the main result of the current work. The comparison of the third and fourth columns shows the way a compact but not very focal modest increases in spectral power before KCs relative to NREM2 core periods (in the third column) shrink in both the spatial and frequency domain to the prominent and highly focal increases in the alpha band (third figurine of fourth column). Comparing figurines within the third column only, it is evident that the single highly focal prominent increases before KCs in the alpha band (third figurine), persist and remains the only prominent increase in the adjacent theta and low sigma bands (second and fourth figurines) for three of the four subjects. Note that different groups of three subjects could be identified in the different figurines, i.e., a different subject may be the one that has not reached prominent level in the theta from the one that did not reach the prominent level for the low sigma band.

### Spectral Patterns of Key Areas in Distinct POIs and First Conclusions

In the Results section we described increases in spectral power from the NREM2 core levels within the rostral and dorsal ACC in the POIs before spindles and KCs. We identified common precursors of spindles and KCs in dcACC2 in the delta band that become distinct in the border band between delta and theta. In the 2 s preceding the start of either spindles or KCs, only one area, the dcACC1, survived at the threshold of *p* < 0.00001: only in the pre-KC but not the pre-spindle period, and only in two adjacent and overlapping frequency bands (high theta and low alpha). The two focal areas dcACC1 and dcACC2 seem to play key roles for KCs and spindles, respectively, but these roles must be seen as the apex of specialization of a unified system (the brain) with areas across the ACC and more dorsal and rostral areas involved in a very specific way around the KC and spindle generation.

Table 3 shows that such a common precursor is the active inhibition (increase in the spectral power in the delta band) in dcACC2 and the immediately dorsal part of SMA (anterior side of SMA). Before spindles but not before KCs a similar active inhibition is evident in rACC and pre-SMA. The opposite (active inhibition before KCs but not before spindles) is seen in dcACC1, the area showing prominent increase in higher frequencies and located where the only area in the brain from where electrical stimulation (in awake state) generates KC-like response (Voysey et al., 2015).

During spindles the changes remain modest and focal, with some prominent and very focal activations in the alpha and low sigma bands in dcACC adjacent dorsal midline cortex and in high sigma band in posterior cortex as described in some detail in Ioannides (2017). During KCs the activity is prominent in the entire ACC and adjacent areas in the delta, theta and alpha bands. These results are consistent with very different operations taking place during spindles and KCs. During spindles the process is finely tuned and precise operations seem to take place as has already been discussed earlier (Ioannides et al., 2017; Ioannides, 2018). A very different picture emerges during KCs: Prominent power increases are seen in the frequencies associated with active inhibition (delta) and with cognitive function (theta and higher bands). Furthermore, modest increases in activity are identified in low and high gamma band (see example in Figure 5). These results are consistent with a system running close to a transition, befitting the Janus nature attributed to KCs, a damping effect to promote sleep and an arousing effect for more cognitive tasks that may lead to awakening (Jahnke et al., 2012; Halász, 2016). The findings above, collected from both this work and those previously reported (Ioannides et al., 2009, 2017), converge to the following conclusions:

• The KCs, like spindles, should be considered as the “end product” of a long sequence of processes. This sequence starts with sleep onset in distinct neural networks but becomes most prominent at specific sleep stages, at specific periods before and during KCs and at specific nodes of the brain networks. During this long sequence distinct parts of the ACC play key roles (shown together in Figure 6) with changes in spectral power at the very specific frequency bands describe above and listed in Table 3.
• The only prominent change in the before POIs is the focal increase of high theta and low alpha spectral power before KCs. This focal increase is in the caudal end of the rostro-caudal part of the anterior cingulate cortex (dcACC1). This area coincides with the area identified recently as the only location where electrical stimulation (while awake) evokes KC-like responses.
• The ACC is involved in environmental and internal monitoring - consistent with a sentinel role. As discussed below, analysis of data from related experiments, for conflict and environment monitoring, identified areas within a few millimeters of the rACC and the two dcACC areas, and the wide ACC areas between them.
• A modest increase is identified in the delta band in the dcACC2 and SMA proper that is common to both spindles and KCs (white outline in rightmost figurine of Figure 3. This likely represents a common active inhibition before spindles and KCs. This is probably part of mechanism preventing movement by actively inhibiting the SMA and dcACC2, two areas directly connected with movement initiation (Passingham, 1995; Picard and Strick, 2001). Apart from this common change, the changes in spectral power before KCs were different than those for spindles, either in terms of frequency or in terms of location, but often sharing a common border, see for example how the wider activation in the delta band (center frequency at 3.2 Hz, range from 1.6 to 4.8 Hz) with the common area marked by the white outline, separates in the next band (center frequency at 4.8 Hz, range from 3.2 to 6.4 Hz), into distinct but adjacent increases for spindles and KCs.
• Prominent increases relative to NREM2 core periods are focal in the pre-KC period and extended but well circumscribed (not global) during KCs, as can be seen in Figures 2–4 of this work and Figures 9–11 in Ioannides (2017).

### The Distinct Spectral Signatures Before and During KCs Across Studies

The key areas involved in KC generation are dcACC1, sgACC, and pre-SMA. Before and during KCs, spectral increases are identified in dcACC1 for all bands with centers from 3.2 to 11.2 Hz and for higher frequency bands for the POI during KCs. During the pre-KC period, modest increases are identified for bands with centers from 8 to 11.2 Hz in the pre-SMA. In sgACC, only a modest increase is identified in the pre-KC period and only in one band with center at 8 Hz. During KCs, prominent changes are identified from 3.2 to 12.8 Hz (a modest one in the next band with center at 14.4) in both sgACC and pre-SMA, with a modest increase at 16 Hz only for sgACC. The spectral signature for spindles and KCs are distinct and they provide hints for their possible roles. For KCs the coexistence of delta increases (active inhibition) with increases at higher frequencies (increase cognition) suggest a struggle between external influences and the need to decide whether to wake up or not, and sleep promoting activity, consistent with the proposed sentinel role of KCs (Halász, 2016; Blume et al., 2018). Our analysis adds to the existing literature the identification of this Janus-like behavior of KCs, not only during the main event, but also in the pre-KC period in the frequency range from delta to sigma bands, consistent with recent findings using EEG (Blume et al., 2018).

The results described above and in the previous sub-sections and the first conclusions drawn from them are in excellent agreement with recent literature as the examples of the previous paragraph show. Studies with intracranial electrodes are particularly supporting, including the only study where stimulation of a specific brain area (the dcACC) was causally related to KC-like activation (Voysey et al., 2015). There seemed to be just one apparent exception: in a recent paper (Mak-McCully et al., 2015), it was reported that “*Locally generated KCs were found in all sampled areas, including cingulate, ventral temporal, and occipital cortices. Surprisingly, KCs were smallest and occurred least frequently in anterior prefrontal channels.*” This statement stands in sharp contradiction to the identification of focal initiator of KCs in dcACC in both our study and in the recent intracranial stimulation study (Voysey et al., 2015). A closer examination of the details of Mak-McCully et al. (2015) reveals that the hard facts are actually in full agreement with our findings: the KCs identified by human experts had a local generation in 76% of the cases. The global distribution was identified after more “KC-like” phenomena were included “*using an automatic procedure to look for KC-like activity in the channels where KCs were not manually marked, at times when a KC was manually marked in at least one channel. Each channel was band-passed with a zero phase-shift filter from 0.2 to 5 Hz before the detection of KC-like activity. For each channel, a KC template was created by averaging over the manual KC detections from −350 to* + *650 ms around the peak. A sliding inner product was then performed between this template and a 1.2 s window when a manual KC occurred in at least one channel.*” Filtering with low pass set to 5 Hz eliminates the higher frequencies, e.g., upper theta/low alpha component (Kokkinos et al., 2013) while averaging from the peak negativity further biases the outcome against the other components (positivities before and after the negativity) which have variable time relation with the peak negativity. The combined effect will be a selection of activity near the peak negativity and therefore correspond to the periods “during KCs” of our studies, where we also found widespread activations in the delta band, as seen in Figure 4 of Ioannides (2017) and Figure 4 of this paper and in Table 3. Further, our current study shows that these widespread activations during KCs are seen in similar areas in the theta and alpha bands. The key finding of the present study is that in the 2 s preceding the KC, there is a prominent (*p* < 0.00001) increase in the spectral power in the high theta and low alpha bands over the NREM2 core period confined to dcACC1. As best as one can judge from the published figures (Voysey et al., 2015), this is exactly the area identified as the only area (from the ones where electrodes were placed) that when stimulated causes a KC-like event.

### Consistency of the Results and Putative Roles of the KC Generator in ACC

The ACC has been implicated in NREM slow wave generation (Dang-Vu et al., 2008) while it has also been shown to precede sleepwalking episodes (Januszko et al., 2016) and NREM parasomnia arousals (Terzaghi et al., 2012). To advance our understanding of ACC’s role however, it is worth integrating relevant evidence from the awake condition as well, beginning by noting that the KC-like event “caused” by stimulation of the dcACC has been seen during awake state (Voysey et al., 2015). Paus (Paus, 2001) emphasized three key roles of ACC in behavior (a) in motor control through dense projections to the motor cortex and spinal cord [see also (Hoffstaedter et al., 2013)], (b) in cognition through reciprocal connections with the lateral prefrontal cortex [see also (Dehaene, 2018)] and (c) in arousal/drive state through extensive afferents from the midline thalamus and brainstem (mainly noradrenergic and dopaminergic nuclei [see also (Vogt and Gabriel, 1993)].

The ACC is generally known to be involved in saliency detection and environmental monitoring, with a rough division into rostral ACC and dorsal ACC sectors corresponding to a rough segregation of emotional (Drevets et al., 2008) and cognitive (Steele and Lawrie, 2004; Taylor et al., 2007) functions, respectively. There are numerous studies demonstrating changes in the ACC with fine experiments attempting to disentangle specific parts of ACC excited differentially by specific tasks, ranging from conflict monitoring in Stroop effect (Pardo et al., 1990), error monitoring (Carter, 1998; Taylor et al., 2007) and its dependence on awareness (O ’connell et al., 2007) and adjustments in control (Kerns et al., 2004). A positron emission tomography study using theory of mind (ToM) tasks showed a reduction of dcACC activity when subjects listened to unlinked sentences compared to actual stories and in two nearby activation in the contrast between ToM and physical stories (Fletcher et al., 1995).

The evidence quoted above and that from our present and earlier (Ioannides et al., 2017) studies converge to the conclusion that similar mechanisms with the same key nodes are involved while awake and sleep to monitor the environment with the ACC playing a central role in both conditions. How this monitoring is used might be different: while awake the results lead to actions biased by intentions; during sleep a simpler choice is selected, to continue sleeping and doing what sleep allows doing or wake up to deal with what just happened. Within such an integrated view the present results help us understand the sentinel function in sleep and the potential key role of KC in it. All sleep functions (i.e., restoration, replenishment and especially memory consolidation through synaptic plasticity and reorganization) demand a brain of low energy consumption and non-biased information processing, i.e., a brain not interfered with and not interrupted by stimuli (environmental or internal). However, complete isolation would render an animal vulnerable to predators or homeostatic emergencies. The compromise between such worthiness of sleep and that of being awake in the face of danger is one of the great challenges in biology. A convenient solution to this challenge could be a sentinel type process, fast evaluating the saliency and/or alarm character of internal and external signals and accordingly lead to awakening or sleep maintenance (Jahnke et al., 2012; Halász, 2016). A sentinel brain system must possess at least three capabilities: sensitivity/responsiveness, cognitive evaluation/decision taking as well as sleep restoration/hypnagogic action; and all of them are potentially met by KC:

(a) There are indications of KC reflecting sensory responsiveness and/or signs of unconscious arousal and being accepted as a reactive wave (Halász, 2016), spontaneously emerging, as an accurate representations of late components (> 400 ms) of sensory evoked potentials by any modality stimuli, (Niiyama et al., 1996; Bastuji and Garcia-Larrea, 1999; Cote et al., 2000; Jahnke et al., 2012) as well as stimuli in anterior cingulate gyrus (Voysey et al., 2015). It is intriguing that the sleeping brain is momentarily more responsive to incoming sensory events just preceding a KC (Sallinen et al., 1997) the time of prominent activation of dcACC (see Figures 2B,C), a brain area known to be involved in saliency detection and environmental monitoring (Steele and Lawrie, 2004; Taylor et al., 2007).
(b) There are KC electrophysiological correlates of cognition i.e., fast neural activity of a type – gamma band EEG, but also alpha and theta - known to support cognitive processes (Gross and Gotman, 1999; Başar et al., 2001), required in order to evaluate the saliency of the stimulus. Localized gamma activations have been observed throughout sleep including NREM2 (Ioannides et al., 2009, 2017). Here we demonstrate that gamma activation in the low and high gamma band is seen just prior and during KCs throughout the ACC with only one example shown in Figure 4. In this example, the increase in gamma band power close to 60 Hz in dcACC, just rostral and ventral of the area with prominent high theta and low alpha activations (Figures 2, 3). Interestingly a previous EEG study showed during KCs brief bursts with anterio-posteriorly mobile maximal power and increasing frequency (from high theta to low alpha) (Kokkinos et al., 2013). Consistent with cognitive activity associated with KC are considered its habituation (Bastuji et al., 1995) and its ability to discriminate rare stimuli (Bastuji et al., 1995; Pratt et al., 1999) or stimuli of self-relevance and emotional significance (Perrin et al., 1999; Blume et al., 2017). Importantly, the area where KCs appear to emerge from (ACC) is an area associated with detecting internal or external perturbations (Luu and Pederson, 2004) - further relating KC to a sentinel role.
(c) There is indirect but strong and converging evidence for an hypnagogic (protecting sleep and promoting its maintenance) effect following KC, since the impact of intrusive arousals is lessened and so awareness and awakening is prevented through arousal inhibition, (Wauquier et al., 1995; de Gennaro et al., 2000; Amzica and Steriade, 2002; Colrain, 2005; Halász, 2005; Forget et al., 2011). Also, an increase in KC density has been observed in association with an increase in delta wave leading to slow wave sleep or following a night of fragmented sleep (de Gennaro et al., 2000). A range of different mechanisms have been proposed to describe the KCs sleep promoting role, from the synchronization expected from large and fairly generalized slow negative waves, to the triggering of spindles by KC, to the resetting of brain activity in the transition from Down to Up state (Cash et al., 2009). Any of these mechanisms may theoretically protect sleep.

We conclude that all three demands for a sentinel operation (sensitivity, decision making and restoration of sleeping state) are fulfilled. But, can a tentative synthesis be made? This is a formidable task to tackle, but we think that the appreciation of the fundamental role of dcACC in the emergence of KC (Voysey et al., 2015; Ioannides et al., 2017) points new investigations in specific directions.

### Contribution to Old Debates and Speculations

There is considerable debate about the nature of KCs and whether or not they have local or global generation. There is also a seemingly independent controversy about local or distributed nature of awake state processing of distinct physical and cognitive stimuli and situations and specifically pain, cognition, environmental monitoring through error prediction and negative emotion (Shackman et al., 2011; Jahn et al., 2016). In this subsection we will attempt to use the results for the analysis of spectral activity around spindles and KCs to provide bridges between seemingly diverging conclusions and use the resulting synthesis in the final subsection for airing a falsifiable speculation about KCs and ERN.

We begin by noting that distinct brain networks become substantially disconnected during sleep (Massimini et al., 2005), but from time to time particular networks succeed to communicate and lead to a global event (Gemignani et al., 2015). This large amplitude event, however, in NREM sleep cannot enrich consciousness, as in awake, but instead triggers a massive reset of many networks. One of the mechanisms of such resetting could be an evoked neural bistability, which prompted some to describe the nature of KC, as an isolated DOWN state (Cash et al., 2009). Candidate mechanisms for global brain networks resetting include activation of locus coeruleus (Bouret and Sara, 2005). Although consciousness is lost, some unconscious monitoring seems to reappear after sleep onset (Bush et al., 2000; Luu and Pederson, 2004); a strong evidence for this is the focal alpha and low sigma spectral power increases in rACC and other frontal areas in the core periods of NREM2 compared to those of NREM1 (Ioannides et al., 2017). This highly focal change in activity we interpret as a local move away from down states is augmented around KCs. Our results show that both the pre-KC POI and the POI during KC the spectral power relative to that of NREM2 core period is higher in low (delta) and high (theta, alpha, and sigma) frequencies. The increases in the pre-KC POIs are relatively focal, becoming stronger and expanding more widely in the frontal brain in the POIs during KCs. Furthermore, focal increases are encountered in the gamma band which have similar strength and extend in the pre-KC and during KC POIs as can be seen in the example of Figure 5.

A related point concerns the question of local or global emergence of KCs. It appears that this debate is won by the former, provided high spatial resolution is afforded (Murphy et al., 2012; Pugin et al., 2015; Ioannides et al., 2017; Siclari and Tononi, 2017). Pre-KC activations in ACC and more dorsal medial frontal cortex appear to be a fundamental component of a sentinel system initiating an unconscious cognitive process which allows a safe and sound, uninterrupted sleep. Both the results presented here and the recent intracranial report of stimulation of dcACC evokes (in awake state) KC-like behavior (Voysey et al., 2015) clearly point to a local generation of KC. This, however, is not the complete story. The study of the intact system in our analysis shows that, at least in the pre-KC POI, focal spectral changes preceding the KCs. During KCs the changes in the spectral power below the beta band are widespread, especially in the frontal cortex where widespread increases survive even at the prominent threshold of *p* < 0.00001.

Our results offer an alternative point about the nature of KCs and the debate whether the emergence of KCs is a local or global phenomenon. Combining our findings with the widespread delta activity around KCs found by Mak-McCully et al. (2015), the evidence suggests that the pre-KC POI is characterized by a local departures from an otherwise widespread down state. Furthermore this pattern of pre-KC increase is a continuation of the changes already taking place in the core periods (well away from spindles and KCs) from NREM1 and NREM2. During the pre-KC POI the potentially disruptive event (the KC) is initiated at dcACC1 and propagates to a few other areas leading to a set of confined focal events. Under this interpretation, our results suggest that the KC itself (i.e., during KC POI) is not a pure down state, but a bistable disturbance with strong components with spectral power in both the delta band, but also in the higher frequencies. This is very likely a reflection of the Janus face behavior reported by many authors (Jahnke et al., 2012; Kokkinos et al., 2013; Halász, 2016; Blume et al., 2018). This strong statement is made with some reservation because of the low number of subjects in our study. More work with detailed analysis of the coupling between areas, including cross frequency and phase amplitude coupling could provide some additional support and also clarify the precise role of the increases in the transition frequencies (theta band) that now are provisionally seen as the upper bound of the sleep promoting facet of KC (Kokkinos et al., 2013; Gonzalez et al., 2018).

The last key discussion point deals with the functional parcelation of the dorso-caudal part of ACC and the premotor cortex directly dorsal to it; this midline brain area is central to our discussion and it is represented in Figure 6 by the quartet of four ROIs: dcACC1, dcACC2, SMA and pre-SMA. The reviews by Picard and Strick (Picard and Strick, 1996, 2001) brought together primate microstimulation, human brain imaging (PET and fMRI), physiology and connectivity analysis, attempting to separate distinct of cytoarchitectonic areas on the medial wall related to motor planning and control. Two areas, the SMA-proper (as we also define it), located just rostral to the motor cortex and the cingulate area ventral to it (our dcACC2) were found to have behaviors consistent with direct motor role (e.g., eliciting a time-locked motor output when stimulated), while the pre-SMA appeared concerned more with the selection aspect of motor acts and having no obvious time locked motor output. The same part of the medial wall of the brain figured prominently in a series of other studies using tasks modulated by the level of conflict, error detection, and error prediction. These tasks seemed to elicit a characteristic evoked response potential in the EEG known as error related negativity (ERN) (Bush et al., 2000), apparently generated in the ACC (Herrmann et al., 2004; Dehaene, 2018). More recent intense efforts to understand the functional organization of the SMA, pre-SMA and the rostral and dorsal ACC boils down to the question: is the processing of emotion, pain and cognitive control segregated in distinct subdivisions or are these integrated in a common region? A series of studies, reviewed in Shackman et al. (2011) convincingly demonstrated an overlap of areas identified in tasks involving negative affect, pain and cognitive control. Shackman et al. (2011) reported maxima in separate analysis for each category localized very close to the areas identified in our analysis:

• for negative affect at (TC: −2, 10, 38; within 3 mm of dcACC1) and at (TC: −2, 30, −2; within 3 mm of sgACC).
• for pain at (TC: −2, 0, 44; within 2 mm of dcACC2) and at (TC: −2, 0, 44; between rACC (within 12 mm) and sgACC (within 15 mm).
• for cognitive control at (TC: 0, 12, 42; within 3 mm of dcACC1 and 7 mm of pre-SMA).

A “conjunction map” pooling the pairs of, or all three unitary maps, shows the voxels that were consistently activated across the two or three domains. The resulting conjunction point coincided with the point identified in the unitary map for cognitive control, at (TC: 0, 12, 42) less than 3 mm away from our dcACC1. The cluster maximum for the conjunction between pain and cognitive control (without negative affect) is at (TC: 0, 9, 39) the closest at 1.4 mm away from dcACC1.

In a second study (Jahn et al., 2016), effects of pain, conflict and prediction error (PE) was independently manipulated in a single experiment, allowing a direct comparison of pain and cognitive processing within subjects. The study reported a double dissociation with pain eliciting activations ventral to the cingulate sulcus and cognitive effects localizing more dorsally within the dorsal ACC and spreading into the pre-supplementary motor area, thus supporting the reverse inference meta-analysis of Lieberman and Eisenberger (2015). In general, the cluster maxima, representing foci of significant activations for well segregated properties identified were not as close to the areas identified in our study as the ones identified in Shackman et al. (2011); the closest to our key areas in the (Jahn et al., 2016) study was the cluster maxima for the main effect for Pain at (TC: −2, 28, 13; within 6 mm of rACC), with the other two main effects producing cluster maxima closest to dcACC1 (for PE at TC: −3, 22, 44; within 13 mm) and for Conflict at (TC: 0, 21, 40; within 11 mm).

Spindles and KCs are special processes established by evolution, the greatest experiment of all, to distinguish mechanisms that although starting from a common progenitor (the core state of NREM2) evolve to satisfy different requirements. The identification of distinct foci in the diverse studies discussed above that are very close to the main foci we have identified in our studies provides some supportive evidence. The closeness is particularly impressive in the case of meta-analysis that seek to identify common activations across studies with widely different content (Shackman et al., 2011): nearby foci were identified to each one of the six areas we have identified related to the POIs before and during spindles and KCs. In our view, this consistency is almost inevitable, given the fact that the areas we identify correspond to distinct cytoarchitectonic areas, as already demonstrated for the division of the pre-motor cortex into SMA and pre-SMA (Passingham, 1995; Picard and Strick, 1996, 2001) and the division of the cingulated as already implied by the existence of at least three distinct somatotopic maps (Picard and Strick, 1996, 2001). Significantly, the area identified as the cluster peak of the treble conjunction for negative affect, pain and cognitive control (Shackman et al., 2011) is the same as the one identified in the unitary analysis for cognitive control and only 3 mm away from dcACC1, the highly focal activation that we have identified as the prominent activation in the pre-KC area in the high theta and low alpha range and the only area from where KC-like activity can be elicited in awake state (Voysey et al., 2015). The KC emerges as the general mechanism performing the sentinel duty for whatever disturbance might arise during NREM2 and this can be best described either as a deviation from predicted homeostasis, pain, too strong affect, strong and unexpected external stimulus. It is therefore to be expected that the areas closely related to the KC will correspond to the areas identified in both the unitary and conjunction maps of the (Shackman et al., 2011) study.

We offer a speculation that emerges from our results that can serve as a guiding hypothesis for future experiments with the same subjects in awake state and sleep. This speculation can be supported, modified or falsified by the future experiments we propose below. We suggest that the KC is the sleep side of the well-studied error related negativity (ERN) that is usually studied in awake state. ERN and KC share a neurophysiological (negative wave) and localization (ACC) characteristics. A recent review of the history of ERN studies (Dehaene, 2018) concludes that ACC is a key area of ERN generation and it is the place where an internal comparison of two signals is made: an unconscious representation of the ongoing action and a conscious representation of the intended one. Although these studies are performed in awake state, ERN may not be unrelated to sleep, since sleep deprivation causes inefficiencies in error-monitoring, as reflected by a reduction in amplitude of the ERN (Fueggle et al., 2018). This speculation opens up a new question. Given that a sentinel role is also encountered for spindles, is there any related spindle activity that might relate to an ERN-like activity during awake state? The way the four areas at the center of this investigation, dcACC1, dcACC2, Pre-SMA and SMA-proper are activated before and during spindles and KCs provide some hints. Although it seems that these four areas are involved in both spindles and KCs, it seems that dcACC1 and pre-SMA are more related to KCs while SMA and dcACC2 are more related to spindles. The earlier studies for the roles and connections of these areas (Picard and Strick, 1996, 2001; Ruan et al., 2018) suggest that there may be a separation between motor specific aspects and learning. The KCs may be more involved in the detection of the need to act and the decision as to whether action should be taken (continue sleeping or wake up) while spindles may be more involved in the enacting of motor acts as part of their role in learning.

### Limitations

The small number of subjects (four) is a possible limitation of this study and further studies with more subjects are needed to remove concerns about the generalizability of the results to other subjects. However, the methodology adopted here allows detailed tomographic and statistical analysis of individual subject data, producing very robust results. For the analysis of each subject stringent statistics were applied to reveal commonalities in all four subjects at two statistical thresholds.

A further limitation is that while KCs can also be elicited by sensory stimuli (Laurino et al., 2014), the present study examines only spontaneous KCs. Furthermore, our work indicates the main nodes of the circuits associated with NREM features, the times of their activation and the spectral content, but not the interactions between nodes and the overall properties of the network.

Another limitation of our study is that there was no control of what activities our subject did before sleep. Studies are urgently needed that, like the recent ones reported by the team of Schabus, which relate sleep patterns to different ages (Hoedlmoser et al., 2014) and the subject’s activity and learning before sleep (Jegou et al., 2019). In our review of related literature we avoided references to mood disorders that are clearly relevant, since these need to be studied separately in awake state and sleep. Nevertheless the relevance is obvious since in many such clinical conditions have shown abnormalities in the frontal half of the cingulate and surrounding cortex (Dolan et al., 1995; Mayberg, 1997; Etkin, 2010).

### Future Outlook

The results outlined in this work add to the growing body of recent sleep research focusing on better understanding of spindles, KCs and slow waves using intracranial recordings (Mak-McCully et al., 2015; Voysey et al., 2015) multichannel EEG (Bernardi et al., 2018; Blume et al., 2018). In this and other of our recent works (Ioannides et al., 2009, 2017; Ioannides, 2018) we relied on the ability of MEG to extract detailed tomographic estimates of activity Focusing almost exclusively on sSPM analysis. However, as can be seen in Figure 7, the availability of robust sample by sample estimates of changes in activity in key ROIs opens up new options for detailed time frequency analysis, connectivity analysis and for comparing slow oscillations with single and multiple KCs at unprecedented detail.

Another important way forward is through new experiments that combine at the level of individual subject awake state experiments and whole night sleep studies. Also, from an operational point of view, error in the comparison between ongoing and intended action, is not fundamentally different from saliency detection. We therefore propose that MEG (or EEG) experiments should be performed in awake state and sleep using the same subjects. In the awake state protocols that elicit ERN components should be used, e.g., Go/NOGO experiments with stop signal conditions. The sleep experiments should allow the subjects to reach at least NREM2 so enough spindles and KCs should be collected. Ideally somatosensory and auditory stimulation during NREM2 should also be used. It will also be useful to add stop signals before an expected GO stimulus as well as after. The prediction is that increase activity will be identified in the key areas identified in our study, especially dcACC1, pre-SMA and sgACC for the ERN and before KCs. Activation of dcACC2, SMA and rACC related to ERN could help us clarify not only their role in ERN but their contribution before and especially during spindles. If ERN and KC (and aspects of spindles) are indeed manifestations of the same basic process (e.g., related to impulsivity and response to environmental change) in awake state and sleep, respectively, we would expect the relative contribution from each of these areas to vary from subject to subject but be more similar for KCs and spindles and ERN within each subject. If evoked KCs are available too, then one will be able to map the changes in awake state and sleep from the related sensory areas to the putative ERN and KC generators and compare their respective evolutions, thus identifying where and how the two evolutions differ.

## Data Availability

The raw data supporting the conclusions of this manuscript will be made available by the authors, without undue reservation, to any qualified researcher.

## Ethics Statement

RIKEN’s (the institution where the experiment was conducted) ethics committee approved the study, and all the subjects gave their informed written consent after all procedures were explained to them before the experiment.

## Author Contributions

AI conceived, initiated, and directed the study, for both the experimental phase at the RIKEN BSI and the analysis in Cyprus. All authors contributed to the initial experimental planning, discussed the meaning of the findings, and wrote the manuscript. AI adapted the analysis methods to specific needs of the study and together with LL performed the data analysis in Cyprus.

## Conflict of Interest Statement

AI and LL were employed by the company AAISCS. The remaining author declares that the research was conducted in the absence of any commercial or financial relationships that could be construed as a potential conflict of interest.

## Footnotes

1 http://sprout022.sprout.yale.edu/mni2tal/mni2tal.html
